# Novel *MAML2* Fusions in Human Malignancy †

**DOI:** 10.3390/cancers17193146

**Published:** 2025-09-27

**Authors:** Takefumi Komiya, Kieran Sweeney, Chao H. Huang, Anthony Crymes, Emmanuel S. Antonarakis, Andrew Elliott, Matthew J. Oberley, Mark G. Evans

**Affiliations:** 1 Division of Hematology and Oncology, Penn State College of Medicine, Hershey, PA 17033, USA; 2 Caris Life Sciences, Phoenix, AZ 85040, USA; ksweeney@carisls.com (K.S.); aelliott@carisls.com (A.E.); moberley@carisls.com (M.J.O.); mevans@carisls.com (M.G.E.); 3 Medical Oncology Division, Department of Internal Medicine, University of Kansas Medical Center, Kansas City, KS 66103, USA; chuang2@kumc.edu; 4 Department of Medicine, Keck School of Medicine, University of Southern California, Los Angeles, CA 90007, USA; acrymes@dhs.lacounty.gov; 5 Division of Hematology, Oncology and Transplantation, Masonic Cancer Center, University of Minnesota, Minneapolis, MN 55455, USA; anton401@umn.edu

**Keywords:** MAML2, YAP1, ATXN3

## Abstract

Background: Oncogenic fusions of *MAML2* with *CRTC1*, *CRTC3*, *YAP1*, and *NR1D1* retain the *MAML2* transactivating domain (TAD) and are believed to drive aberrant gene transcription. While the oncogenic roles of these known fusions have been established, we aimed to identify novel *MAML2* fusions across a range of human malignancies. Methods: DNA and RNA sequencing were performed on tumor samples submitted to Caris Life Sciences. *MAML2* fusions were identified from RNA transcripts and filtered to include only known pathogenic fusions or recurrent, in-frame fusions containing a C-terminal *MAML2* TAD. Fusion burden was defined as the number of unique fusion isoforms per sample. Results: Among 180,124 tumor samples, 143 specimens harbored *MAML2* fusions with a *MAML2* TAD: >50% of specimens harbored known fusions, but novel fusions with *MTMR2* (31/143), *SESN3* (11/143), *CCDC82* (6/143), *FAM76B* (4/143), and *ATXN3* (3/143) were also identified. Compared to the known fusions, the novel fusions generally had lower expressions (median: 8 vs. 13 junction reads/sample, *p* = 0.0064), higher fusion burdens (median: 6 vs. 2 unique fusion isoforms/sample, *p* < 0.0001), more frequent *TP53* co-mutations (80% vs. 11.5%, *p* < 0.0001), and no clear association with the tissue of origin. Excluding *ATXN3::MAML2*, the novel fusion partners were located near *MAML2* in the genome, likely arose from duplications or deletions, and occurred in samples harboring concurrent mutations. In contrast, *ATXN3::MAML2* arose via interchromosomal translocation, occurred in samples with a low fusion burden, and was not associated with TP53 mutations. Conclusions: We identified novel *MAML2* fusion partners, most of which likely represent passenger alterations, possibly arising from genomic instability or impaired p53 function. However, *ATXN3::MAML2* fusions, previously reported in a pre-cancerous pancreatic disease case, may represent a pathogenic alteration warranting further investigation.

## Simple Summary

Many gene fusions have been implicated in the development and progression of human cancers. Our group and others have identified specific gene fusions involving the *MAML2* gene in human cancers, and we were interested in searching for additional novel *MAML2* fusions. Our analysis with the Caris Life Sciences molecular database demonstrated that most of the novel fusions were associated with increased fusion burden and genomic loss of heterozygosity, which are potentially suggestive of genomic instability, except for *ATXN3::MAML2* which warrants further investigation.

## 1. Introduction

*MAML2* (Mastermind-like 2) is a family member of Mastermind genes that are primarily involved in regulation of the NOTCH signaling pathway. *MAML2* activates downstream signaling by binding intra-cellular NOTCH and consists of five exons in which the NOTCH binding domain and transcriptional activation domain (TAD) are located at the N-terminal and C-terminal portions, respectively [1].

Our group was involved in the discovery of a *CRTC1::MAML2* fusion gene, which resulted from a t (11; 19) (q14–21; p12–13) translocation in mucoepidermoid carcinoma (MECT) [2]. It typically develops in the parotid gland but can occur in other organs, such as the lungs [3]. The fusion is detected in more than 50% of MECT cases, regardless of primary location, and inhibition of the *CRTC1::MAML2* fusion or its downstream targets suppresses cell growth in both in vitro and in vivo models [4,5,6], supporting the notion that *CRTC1::MAML2* is a specific driver oncogene in MECT. Further studies also found *CRTC3::MAML2* fusions in MECT. Both fusions retain the N-terminal CREB binding domain of *CRTC1/3* and the C-terminal TAD of *MAML2* and drive oncogenesis via aberrant expression of *CREB* target genes [7,8,9].

Studies including the one by Valouev A et al. reported frequent in-frame *YAP1::MAML2* fusions in nasopharyngeal carcinoma, metaplastic thymoma, poroma, malignant undifferentiated sarcoma, ependymoma, and others [10,11,12,13,14]. *YAP1* is a transcriptional coactivator involved in the regulation of the Hippo and Wnt pathways [15]. *YAP1::MAML2* fusions retain the N-terminal TEAD-binding region of *YAP1* and the C-terminal TAD of *MAML2* and have been shown to interact with TEAD transcription factors to activate the *YAP1* transcriptional program [16].

In addition to *CRTC1/3* and *YAP1*, other *MAML2* fusion partners such as *CXCR4* in chronic lymphocytic leukemia, *KMT2A* in thymoma, and C11orf95 and ZFTA in ependymoma have been reported [17,18,19,20,21]. The *NR1D1* partner has also recently been described as an emerging epithelioid and spindle cell sarcoma subtype [22]. All reported fusions retain the *MAML2* TAD and do not activate NOTCH signaling due to loss of the C-terminal NOTCH binding site, suggesting that *MAML2* fusions activate the downstream signaling of the partner gene rather than the NOTCH pathway. Our study aimed to identify additional previously undescribed fusion partners of *MAML2* across a range of human malignancies, detected by whole transcriptome sequencing, in which we identified new oncogenic alterations that could be amenable to targeted therapy in cancer subtypes.

## 2. Materials and Methods

### 2.1. Compliance Statement

This retrospective study was reviewed by the institutional review board at Penn State Health and was designated exempt from human subject research. The study was also conducted under Caris Life Sciences’ Research Data Banking protocol, which was reviewed and granted IRB exemption by the WCG IRB. The study adhered to the ethical guidelines of the Declaration of Helsinki, the Belmont Report, and the U.S. Common Rule.

### 2.2. DNA Sequencing

Genomic DNA was isolated from formalin-fixed paraffin-embedded (FFPE) tumor samples and sequenced using the NextSeq or NovaSeq 6000 platforms (Illumina, Inc., San Diego, CA USA). Tumor enrichment was achieved prior to sequencing by harvesting targeted tissues using manual microdissection techniques. A custom SureSelect XT assay (Agilent Technologies, Santa Clara, CA USA) was used to enrich 592 whole-gene targets for the NextSeq sequenced tumors. Over 700 clinically relevant genes at high coverage and read depth were used for NovaSeq sequenced tumors, along with another panel designed to enrich another 20,000+ genes at lower depth. Variants were detected with an average sequencing depth of coverage of >500, an analytic sensitivity of 5%, and >99% confidence based on allele frequency and amplicon coverage. Identified genetic variants were interpreted by board-certified molecular geneticists and categorized according to the American College of Medical Genetics and Genomics (ACMG) standards. Only pathogenic and likely pathogenic mutations were counted when determining the mutation frequencies of individual genes. The copy number alteration (CNA) of each exon was determined by calculating the average depth of the sample along with the sequencing depth of each exon and comparing the calculated result to a pre-calibrated value.

### 2.3. Whole Transcriptome Sequencing

mRNA was sequenced using the Illumina NovaSeq platform (Illumina, Inc., San Diego, CA, USA) and Agilent SureSelect Human All Exon V7 bait panel (Agilent Technologies, Santa Clara, CA, USA). RNA was isolated from formalin-fixed paraffin-embedded (FFPE) tumor samples that underwent pathology review to assess tumor content and size; at least 10% of tumor content in the area for microdissection was required for enrichment and extraction of tumor-specific RNA. RNA extraction was performed using a Qiagen RNA FFPE tissue extraction kit. An Agilent TapeStation was used to determine RNA quantity and quality. Synthesized and purified cDNA targets were hybridized to biotinylated RNA baits; a post capture PCR reaction was used to amplify the bait–target complexes. Resultant libraries were quantified and normalized, and the pooled libraries were denatured, diluted and sequenced. *GRCh37/hg19* was the reference genome. The test had ≥97% Positive Percent Agreement (PPA), ≥99% Negative Percent Agreement (NPA) and ≥99% Overall Percent Agreement (OPA) with a validated comparator method. Gene fusions were identified from WTS data using STAR-Fusion.

### 2.4. Loss of Heterozygosity

Genomic loss of heterozygosity (LOH) was determined by splitting the 22 autosomal chromosomes into 552 segments (2–6 Mb in size) and calculating the LOH of single nucleotide polymorphisms (SNPs) within each segment. Student’s *t*-test was used to compare SNP distances of each region to control distances of a non-LOH reference (NA12878; RRID:CVCL_7526). A region was called positive if the average distance was larger than 0.15 Mb and the corrected *p*-value was less than 0.02. Segments spanning ≥ 90% of a whole chromosome or chromosome arm and segments not covered by the SNP backbone and the WES panel were excluded from the calculation. Samples were called genomic LOH High (LOH-H) if ≥16% of all 552 segments had LOH or Low if <16% of all 552 segments had LOH.

### 2.5. Statistical Analysis

Statistical analysis was performed in Python (v.3.10.4) using the Numpy (v.1.23.3), Pandas (v.1.5.1), and Scipy (v.1.12.0) packages. Significance was calculated using Chi-Square, Fisher’s Exact, or Mann–Whitney-U tests.

## 3. Results

### 3.1. Overview of Fusions

Among 180,124 pan-cancer tumor samples with DNA and RNA sequencing results, 509 samples (0.3%) harbored a *MAML2* fusion. A total of 169 fusions remained after filtering the fusions to retain only those that preserved the C-terminal TAD of *MAML2* (Table S1). After further filtering to focus only on recurrent (occurring in ≥3 patients) or known pathogenic or likely pathogenic (P/LP) fusions, a total of 143 fusion-positive tumor samples remained (Table 1). The fusions included known P/LP fusions of *MAML2* with *CRTC1/3*, *YAP1*, and *NR1D1* and unclassified fusions involving *MTMR2*, *SESN3*, *CCDC82*, *FAM76B*, and *ATXN3*.

*CTRC1::MAML2* and *YAP1::MAML2* were the most common P/LP fusions, and *MTMR2::MAML2* was the most common unclassified fusion. Excluding *YAP1::MAML2*, which arose via inversion, the known P/LP fusions featured partner genes on chromosomes other than *MAML2* that arose via translocation events. In contrast, the unclassified fusions featured partner genes located near *MAML2* on 11q21 and arose from duplications or deletions, except for *ATXN3::MAML2*, which arose from a t (14; 11) translocation.

Functionally, the P/LP fusion partners *CRTC1*, *CRTC3*, *YAP1*, and *NR1D1* were transcriptional activators or repressors. The unclassified fusion partners had diverse functions and no clear role in transcriptional regulation, except for *ATXN3*, a deubiquitinating enzyme reported to control transcription by regulating chromatin structure [23]. The *ATXN3::MAML2* fusions retained most functional domains of *ATXN3*, with C-terminal breakpoints of its PolyQ region.

### 3.2. Clinical Characteristics of Fusion-Positive Patients

Patients with recurrent or *P/LP MAML2* fusions had a median age of 62 years (range: 17–90+) and were 58% female (Table S2). The *CRTC1::MAML2* and *CRTC3::MAML2* fusions occurred primarily in mucoepidermoid carcinomas of the salivary gland or the head and neck; both *NR1D1::MAML2* fusions were detected in sarcomas. *YAP1::MAML2* was appreciated in a relatively broad distribution of cancers; however, a preponderance of these fusion-positive cases arose in the biliary tract (48.7%). Overall, the unclassified *MAML2* fusions had no clear association with the tissue of origin (Table 2).

### 3.3. Expression of MAML2 Fusions

**Figure 1:**
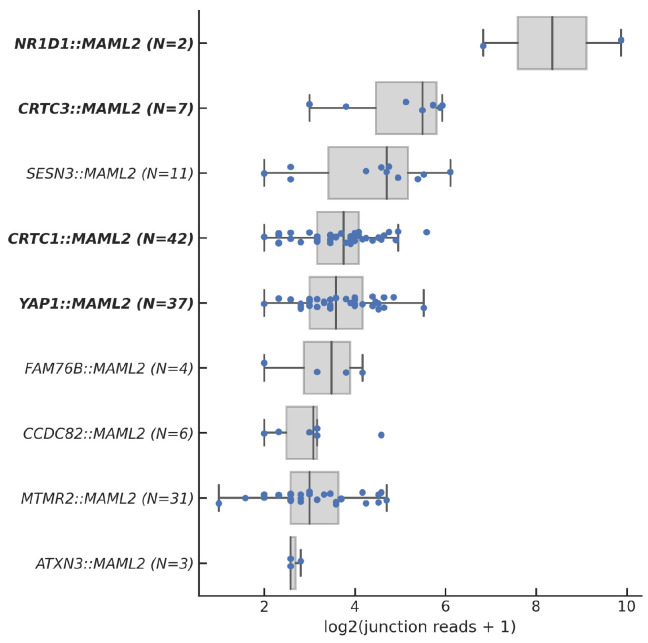
Expression of *MAML2* fusions. Known P/LP fusions are bolded.

We next assessed the expression levels of the *MAML2* fusions (Figure 1). *NR1D1::MAML2*, *CTRT3::MAML2*, and *SESN3::MAML2* were the most highly expressed fusions, while *ATXN3::MAML2* had the lowest expression. Overall, the unclassified fusions had lower expressions than the known P/LP fusions. (8 vs. 13 median junction reads, *p* = 0.0064, Figure S1).

### 3.4. Genomic Landscape of Fusion-Positive Tumors

**Figure 2:**
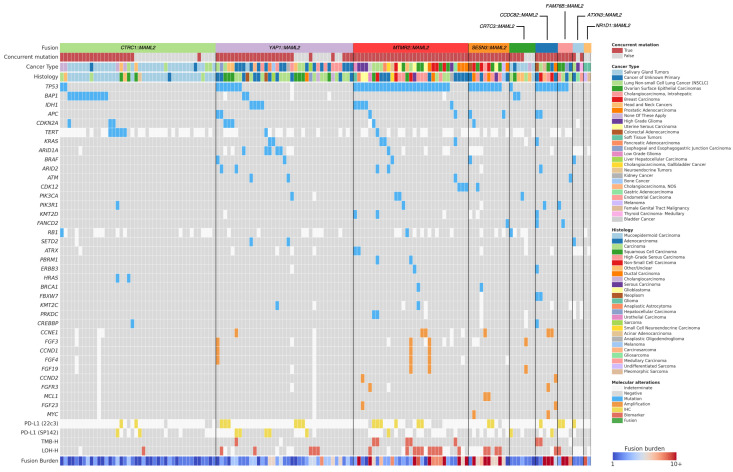
Recurrent genomic alterations and fusion burden in fusion-positive tumors.

**Figure 3:**
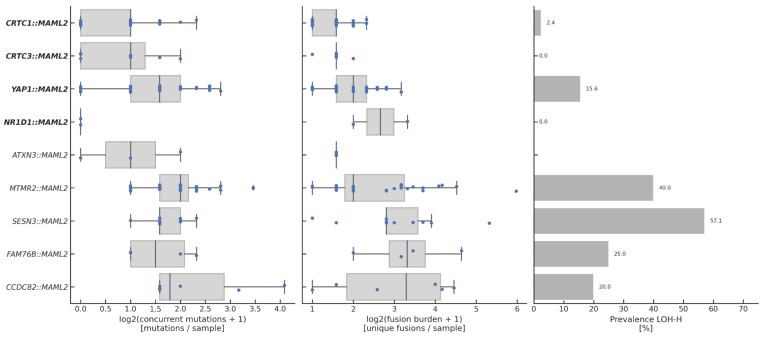
Number of concurrent P/LP mutations per sample (**left**), fusion burden (**middle**), and prevalence of LOH-H status (**right**) by *MAML2* fusion. Fusion burden was quantified as the number of unique fusion isoforms per sample. P/LP fusions are bolded.

The genomic landscape of fusion-positive tumors varied by fusion isoform is shown in Figure 2. Overall, the most common co-alterations were P/LP mutations of *TP53* (*N* = 54), *BAP1* (*N* = 15), and *IDH1* (*N* = 9). Samples harboring unclassified fusions were enriched in P/LP TP53 mutations compared to those with known P/LP fusions (80.0% vs. 11.5%, *p* < 0.0001), though, notably, the *ATXN3::MAML2* samples had none. More generally, samples harboring unclassified fusions tended to have the most concurrent P/LP mutations (Figure 3, left). In fact, with the notable exception of *ATXN3::MAML2*, all samples with an unclassified fusion harbored at least one concurrent P/LP mutation, whereas at least 30% of the samples associated with each known P/LP fusion harbored none (Table S3).

To quantify genomic instability, we also assessed fusion burden and genomic loss of heterozygosity (LOH) (Figure 3, middle and right). Fusion burden was quantified as the number of unique fusion isoforms detected per sample and was positively correlated with LOH (Spearman Rho = 0.60, *p* < 0.0001, Figure S2A). Fusion burden was the highest for fusions involving *FAM76B*, *SESN3*, and *CCDC82* and the lowest for *CRTC1/3* and *ATXN3*. The prevalence of genomic LOH (LOH-H status) was the highest for fusions involving *SESN3*, *MTMR2*, *FAM76B*, and *CCDC82*, but was also notably high among *YAP1::MAML2* samples. Overall, fusion burden was higher in samples harboring unclassified fusions vs. known P/LP fusions (median: 6 vs. 2 fusion isoforms, *p* < 0.0001, Figure S2B), as was the prevalence of LOH-H status (39.0 vs. 7.4%, *p* < 0.0001, Figure S2C).

## 4. Discussion

We retrospectively analyzed a total of 180,124 tumor samples that underwent comprehensive genetic analysis and found 509 specimens with *MAML2* fusions. After filtering out non-recurrent fusions and fusions lacking a *MAML2* TAD, 143 specimens remained.

*CRTC1/3* and *YAP1* were the most common fusion partners of *MAML2*. While previously reported, tumors demonstrating these translocation events may be amenable to targeted therapy. Recent studies indicate that the *CRTC1/3::MAML2* fusions prevalent in mucoepidermoid carcinoma [2,3,8,9] may be susceptible to the effects of *CDK4/6* and EGFR inhibitors [6]. Moreover, several pharmacologic agents are currently undergoing early-phase clinical trials targeting solid tumors with disrupted Hippo pathway signaling [24,25], such as NF2-deleted mesothelioma and tumors harboring *YAP1* fusions [26,27]. These potential therapies could possibly be employed for the *YAP1::MAML2* fusion tumors identified in our study, including those arising in the biliary tract.

Given what is known about the *CRTC1/3* and *YAP1* fusion partners with *MAML2*, our study seeks to determine whether additional, previously unreported partner genes exist and have therapeutic or diagnostic implications. Most of the novel, unclassified fusions have low junction reads, high fusion burden, and frequent co-alterations, notably TP53 mutations. Most of the partner genes were located in the region of the *MAML2* gene on chromosome 11q22. These fusions were seen in tumors arising from a wide variety of organs and did not cluster in any specific disease. These findings suggest that they are potential passenger alterations—products of chromosomal instability, possibly due to impaired p53 function—and do not seem to have any biologic role in tumorigenesis.

Notably, we found three cases featuring in-frame *ATXN3::MAML2* fusions. *ATXN3* is involved in the degradation of misfolding chaperone substrates, transcriptional regulation, and maintenance of protein homeostasis [28]. Depletion of *ATXN3* leads to spinocerebellar ataxia type 3, an age-related neurodegenerative disease. In preclinical models, deletion of the *ATXN3* gene in cancer cells resulted in decreased *YAP1* protein levels and decreased expression of *YAP1* target genes [29], which raise the possibility of targeting the *YAP1/TEAD* transcriptional program as a potential therapeutic strategy for these tumors [27]. Moreover, Lee JW et al. has reported a single case of *ATXN3::MAML2* fusion in a pancreatic intraductal tubulopapillary neoplasm [30]; this novel finding in a pre-cancerous condition could indicate oncogenicity. In particular, our study showed that *ATXN3::MAML2*-positive cases exhibited low fusion burdens and were not always associated with alternate driver mutations, including those involving *TP53*. Ultimately, this novel fusion may warrant further investigation as it occurs in and could be a potential target for human cancers.

### Translational/Clinical Relevance

All previously reported pathogenic fusions involving *MAML2* contain the C-terminus transactivation domain and lose the N-terminus NOTCH binding site. The molecular outcome of these fusions includes activation of downstream pathways/genes of the *MAML2* fusion partner (e.g., *CRTC1/3*, *YAP1*). Selective targeting of *MAML2* fusions requires inhibition of the interaction between the partner gene and its transcriptional targets, emphasizing the importance of individualized therapeutic intervention. Compared to the previously known *MAML2* fusions, our study suggests that rearrangement events with novel gene partners may primarily serve as passenger co-alterations to additional driver mutations, with the exception of *ATXN3::MAML2*, which may be implicated in oncogenesis with further study.

## 5. Conclusions

As an expansion to earlier work [31], we identified novel *MAML2* fusion partners, most of which had no clear role in transcriptional regulation or association with tissue of origin but were associated with increased fusion burden, increased genomic loss of heterozygosity, and impaired p53 function. Further study is needed to determine if the novel fusions are pathogenic alterations or simply passenger alterations associated with genomic instability. In particular, *ATXN3::MAML2* fusions, previously reported in a pre-cancerous pancreatic disease case, may represent a pathogenic alteration warranting further investigation.

## Figures and Tables

**Table 1:** Summary of MAML2 fusions analyzed in study. Known P/LP fusions are bolded.

Fusion	Count	Type	Partner Location	Mechanism	Partner Function
*CRTC::MAML2*	42	Pathogenic	19p13.11	Translocation	Transcriptional activator
*YAP1::MAML2*	37	Pathogenic	11q.22.1	Inversion	Transcriptional activator/repressor
*MTMR2::MAML2*	31	Unclassified	11q21	Duplication	Lipid phosphatase
*SESN3::MAM22.1L2*	11	Unclassified	11q21	Duplication	Leucine sensor
*CRTC3::MAML2*	7	Pathogenic	15q26.1	Translocation	Transcriptional activator
*CCDC82::MAML2*	6	Unclassified	11q21	Deletion	Unknown
*FAM76B::MAML2*	4	Unclassified	11q21	Duplication	Inhibits NF-κB signaling by preventing nuclear translocation of HNRNPA2B1
*ATXN3::MAML2*	3	Unclassified	14q32.12	Translocation	Deubiquitinating enzyme involved in transcriptional regulation
*NR1D1::MAML2*	2	Likely Pathogenic	17q21.1	Translocation	Transcriptional repressor

**Table 2:** Summary of cancer types and histologies by *MAML2* fusion. Known P/LP fusions are bolded.

Fusion	Cancer Type	Histology
*CRTC1::MAML2*	Salivary Gland Tumors: 71.4% (30/42) Lung Non-small Cell Lung Cancer (NSCLC): 9.5% (4/42) None Of These Apply: 9.5% (4/42) Cancer of Unknown Primary: 4.8% (2/42) Head and Neck Cancers: 4.8% (2/42)	Mucoepidermoid Carcinoma: 76.2% (32/42) Carcinoma: 7.1% (3/42) Squamous Cell Carcinoma: 7.1% (3/42) Other/Unclear: 4.8% (2/42) Acinar Adenocarcinoma: 2.4% (1/42) Adenocarcinoma: 2.4% (1/42)
*YAP1::MAML2*	Cancer of Unknown Primary: 40.5% (15/37) Cholangiocarcinoma, Intrahepatic: 21.6% (8/37) Lung Non-small Cell Lung Cancer (NSCLC): 10.8% (4/37) Head and Neck Cancers: 8.1% (3/37) Kidney Cancer: 2.7% (1/37) Liver Hepatocellular Carcinoma: 2.7% (1/37) Esophageal and Esophagogastric Junction Carcinoma: 2.7% (1/37) Pancreatic Adenocarcinoma: 2.7% (1/37) Salivary Gland Tumors: 2.7% (1/37) Cholangiocarcinoma, NOS: 2.7% (1/37) Bone Cancer: 2.7% (1/37)	Carcinoma: 35.1% (13/37) Adenocarcinoma: 27.0% (10/37) Squamous Cell Carcinoma: 18.9% (7/37) Cholangiocarcinoma: 5.4% (2/37) Neoplasm: 5.4% (2/37) Ductal Carcinoma: 2.7% (1/37) Hepatocellular Carcinoma: 2.7% (1/37) Sarcoma: 2.7% (1/37)
*MTMR2::MAML2*	Ovarian Surface Epithelial Carcinomas: 16.1% (5/31) Breast Carcinoma: 12.9% (4/31) High-Grade Glioma: 12.9% (4/31) Lung Non-small Cell Lung Cancer (NSCLC): 12.9% (4/31) Prostatic Adenocarcinoma: 12.9% (4/31) Uterine Serous Carcinoma: 9.7% (3/31) Pancreatic Adenocarcinoma: 3.2% (1/31) Neuroendocrine Tumors: 3.2% (1/31) Low-Grade Glioma: 3.2% (1/31) Head and Neck Cancers: 3.2% (1/31) Gastric Adenocarcinoma: 3.2% (1/31) Cholangiocarcinoma, Gallbladder Cancer: 3.2% (1/31) Bladder Cancer: 3.2% (1/31)	Adenocarcinoma: 25.8% (8/31) High-Grade Serous Carcinoma: 19.4% (6/31) Non-Small Cell Carcinoma: 9.7% (3/31) Carcinoma: 9.7% (3/31) Glioblastoma: 6.5% (2/31) Squamous Cell Carcinoma: 6.5% (2/31) Anaplastic Astrocytoma: 3.2% (1/31) Anaplastic Oligodendroglioma: 3.2% (1/31) Ductal Carcinoma: 3.2% (1/31) Glioma: 3.2% (1/31) Serous Carcinoma: 3.2% (1/31) Small Cell Neuroendocrine Carcinoma: 3.2% (1/31) Urothelial Carcinoma: 3.2% (1/31)
*SESN3::MAM22.1L2*	Breast Carcinoma: 27.3% (3/11) Ovarian Surface Epithelial Carcinomas: 27.3% (3/11) Colorectal Adenocarcinoma: 9.1% (1/11) Endometrial Carcinoma: 9.1% (1/11) Melanoma: 9.1% (1/11) Prostatic Adenocarcinoma: 9.1% (1/11) Uterine Serous Carcinoma: 9.1% (1/11)	High-Grade Serous Carcinoma: 36.4% (4/11) Adenocarcinoma: 27.3% (3/11) Ductal Carcinoma: 18.2% (2/11) Melanoma: 9.1% (1/11) Serous Carcinoma: 9.1% (1/11)
*CRTC3::MAML2*	Salivary Gland Tumors: 71.4% (5/7) Cancer of Unknown Primary: 14.3% (1/7) None Of These Apply: 14.3% (1/7)	Other/Unclear: 42.9% (3/7) Carcinoma: 28.6% (2/7) Mucoepidermoid Carcinoma: 14.3% (1/7) Squamous Cell Carcinoma: 14.3% (1/7)
*CCDC82::MAML2*	Cancer of Unknown Primary: 16.7% (1/6) Colorectal Adenocarcinoma: 16.7% (1/6) Female Genital Tract Malignancy: 16.7% (1/6) Lung Non-small Cell Lung Cancer (NSCLC): 16.7% (1/6) Ovarian Surface Epithelial Carcinomas: 16.7% (1/6) Prostatic Adenocarcinoma: 16.7% (1/6)	Adenocarcinoma: 33.3% (2/6) Non-Small Cell Carcinoma: 33.3% (2/6) Carcinosarcoma: 16.7% (1/6) High-Grade Serous Carcinoma: 16.7% (1/6)
*FAM76B::MAML2*	Ovarian Surface Epithelial Carcinomas: 50.0% (2/4) Cancer of Unknown Primary: 25.0% (1/4) Esophageal and Esophagogastric Junction Carcinoma: 25.0% (1/4)	Carcinoma: 50.0% (2/4) Adenocarcinoma: 25.0% (1/4) High-Grade Serous Carcinoma: 25.0% (1/4)
*ATXN3::MAML2*	High-Grade Glioma: 33.3% (1/3) Lung Non-small Cell Lung Cancer (NSCLC): 33.3% (1/3) Thyroid Carcinoma- Medullary: 33.3% (1/3)	Adenocarcinoma: 33.3% (1/3) Gliosarcoma: 33.3% (1/3) Medullary Carcinoma: 33.3% (1/3)
*NR1D1::MAML2*	Soft Tissue Tumors: 100.0% (2/2)	Pleomorphic Sarcoma: 50.0% (1/2) Undifferentiated Sarcoma: 50.0% (1/2)

## Data Availability

Data will be available upon request to Caris POA program.

## Supplementary Materials

The following are available online at https://www.mdpi.com/article/10.3390/cancers17193146/s1, Figure S1: Expression of known P/LP vs. novel, unclassified *MAML2* fusions. Figure S2: (A) Fusion burden vs. LOH score; fusion burden (B) and prevalence of LOH-H status (C) for samples with known P/LP fusions vs. novel, unclassified fusions. Table S1: Summary of 169 fusions identified with intact C-terminal *MAML2* transactivation domain; the recurrent (*N* ≥ 3) or known pathogenic or likely pathogenic fusions that were the focus of this study are bolded. Table S2: Patient characteristics for samples that harbor recurrent or *P/LP MAML2* fusions (Fusion+) vs. samples that do not (Fusion-).Table S3: Proportion of samples with a concurrent P/LP mutation by *MAML2* fusion.

## Abbreviations

The following abbreviations are used in this manuscript:

IRB	Institutional Review Board
MECT	Mucoepidermoid carcinoma
MAML2	Mastermind-like 2
