# Supplementary material for: Novel MAML2 Fusions in Human Malignancy
Source: Cancers (Basel). 2025 Sep 27;17(19):3146. doi: 10.3390/cancers17193146 (PMC12524005; doi:10.3390/cancers17193146)
Supplement: Supplementary file 1 [file cancers-17-03146-s001.zip › cancers-3854330-supplementary.pdf]

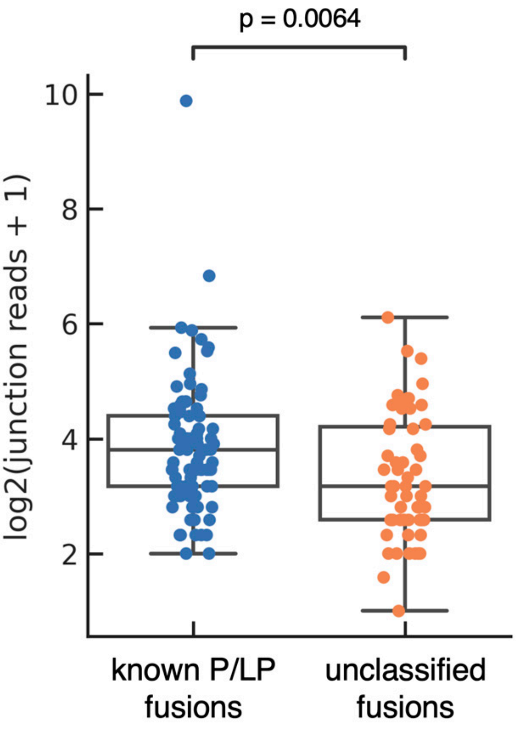

Figure S1. Expression of known P/LP vs novel, unclassified MAML2 fusions.

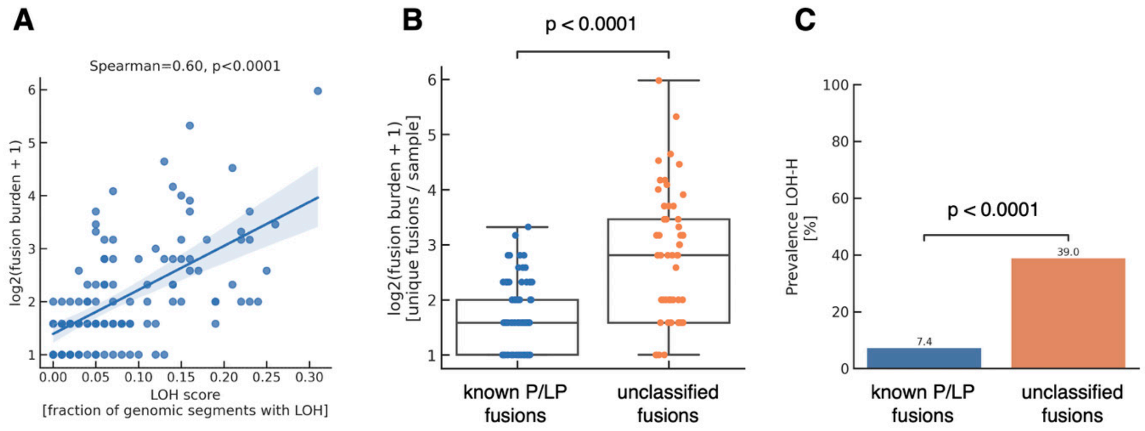

Figure S2. (**A**) Fusion burden vs LOH score. Fusion burden (**B**) and prevalence of LOH-H status (**C**) for samples with known P/LP fusions vs novel, unclassified fusions.

Table S1. Summary of 169 fusions identified with intact C-terminal MAML2 transactivation domain; the recurrent (*N* ≥ 3) or known pathogenic or likely pathogenic fusions that were the focus of this study are bolded.

Fusion	Count
CRTC1::MAML2	42
YAP1::MAML2	37
MTMR2::MAML2	31
SESN3::MAML2	11
CRTC3::MAML2	7
CCDC82::MAML2	6
FAM76B::MAML2	4
ATXN3::MAML2	3
GAB2::MAML2	2
NR1D1::MAML2	2
MARK2::MAML2	2
RDX::MAML2	1
MECOM::MAML2	1
PGM2L1::MAML2	1
PAPSS1::MAML2	1
RPS6KB1::MAML2	1
SLC38A2::MAML2	1
SPECC1::MAML2	1
MED17::MAML2	1
ARHGAP42::MAML2	1
GRM8::MAML2	1
LRP5::MAML2	1
KAT6A::MAML2	1
GPR158::MAML2	1
GPATCH8::MAML2	1
GABRA3::MAML2	1
FCHSD2::MAML2	1
FADS2::MAML2	1
EED::MAML2	1
DDX6::MAML2	1
DDX10::MAML2	1
BIRC6::MAML2	1
ZDHHC5::MAML2	1

Table S2. Patient characteristics for samples that harbor recurrent or P/LP MAML2 fusions (Fusion+) vs samples that do not (Fusion-).

	**Fusion+**	**Fusion-**	**p-value**	**statistic**
**Age**				
Median age (range)	62 (17 – 90+)	66 (0 – 90+)	0.0039	MWU
**Sex**				
Female	58.0% (83/143)	57.2% (103000/179981)	0.8441	Chi-square
Male	42.0% (60/143)	42.8% (76981/179981)		
**Cancer Type**				
Salivary Gland Tumors	25.2% (36/143)	0.5% (953/179981)	0.0005	Fisher's Exact
Cancer of Unknown Primary	14.0% (20/143)	3.4% (6048/179981)		
Lung Non-small Cell Lung Cancer (NSCLC)	9.8% (14/143)	16.7% (30003/179981)		
Ovarian Surface Epithelial Carcinomas	7.7% (11/143)	8.4% (15087/179981)		
Cholangiocarcinoma, Intrahepatic	5.6% (8/143)	1.6% (2798/179981)		
Breast Carcinoma	4.9% (7/143)	7.9% (14178/179981)		
Head and Neck Cancers	4.2% (6/143)	2.3% (4077/179981)		
Prostatic Adenocarcinoma	4.2% (6/143)	3.8% (6754/179981)		
None Of These Apply	3.5% (5/143)	0.5% (951/179981)		
High Grade Glioma	3.5% (5/143)	3.3% (5889/179981)		
Uterine Serous Carcinoma	2.8% (4/143)	1.9% (3441/179981)		
Esophageal and Esophagogastric Junction Carcinoma	1.4% (2/143)	3.1% (5528/179981)		
Soft Tissue Tumors	1.4% (2/143)	1.9% (3482/179981)		
Colorectal Adenocarcinoma	1.4% (2/143)	11.9% (21354/179981)		
Pancreatic Adenocarcinoma	1.4% (2/143)	4.5% (8188/179981)		
Cholangiocarcinoma, NOS	0.7% (1/143)	0.0% (89/179981)		
Thyroid Carcinoma- Medullary	0.7% (1/143)	0.1% (168/179981)		
Bladder Cancer	0.7% (1/143)	3.4% (6137/179981)		
Bone Cancer	0.7% (1/143)	0.4% (641/179981)		
Cholangiocarcinoma, Gallbladder Cancer	0.7% (1/143)	0.6% (1075/179981)		
Female Genital Tract Malignancy	0.7% (1/143)	1.0% (1833/179981)		
Gastric Adenocarcinoma	0.7% (1/143)	1.7% (3117/179981)		
Melanoma	0.7% (1/143)	2.6% (4688/179981)		
Low Grade Glioma	0.7% (1/143)	0.5% (812/179981)		
Liver Hepatocellular Carcinoma	0.7% (1/143)	0.5% (969/179981)		
Kidney Cancer	0.7% (1/143)	1.5% (2703/179981)		
Endometrial Carcinoma	0.7% (1/143)	4.5% (8039/179981)		
Neuroendocrine Tumors	0.7% (1/143)	1.6% (2825/179981)		
Other Cancer Types	0.0% (0/143)	10.1% (18154/179981)		

Table S3. Proportion of samples with a concurrent P/LP mutation by MAML2 fusion.

Fusion	Proportion with P/LP mutation
NR1D1::MAML2	0.0% (0/2)
CRTC1::MAML2	50.0% (21/42)
CRTC3::MAML2	57.1% (4/7)
ATXN3::MAML2	66.7% (2/3)
YAP1::MAML2	70.3% (26/37)
MTMR2::MAML2	100.0% (31/31)
SESN3::MAML2	100.0% (11/11)
CCDC82::MAML2	100.0% (6/6)
FAM76B::MAML2	100.0% (4/4)
